# Trace elements and C and N isotope composition in two mushroom species from a mine-spill contaminated site

**DOI:** 10.1038/s41598-020-63194-2

**Published:** 2020-04-15

**Authors:** Marta Gil-Martínez, Carmen M. Navarro-Fernández, José M. Murillo, María T. Domínguez, Teodoro Marañón

**Affiliations:** 1 Departamento de Protección del Sistema Suelo, Planta, Agua. Instituto de Recursos Naturales y Agrobiología de Sevilla, CSIC, Seville, Spain; 2 Departamento de Cristalografía, Mineralogía y Química Agrícola. Universidad de Sevilla, Seville, Spain

**Keywords:** Microbial ecology, Restoration ecology

## Abstract

Fungi play a key role in the functioning of soil in terrestrial ecosystems, and in particular in the remediation of degraded soils. The contribution of fungi to carbon and nutrient cycles, along with their capability to mobilise soil trace elements, is well-known. However, the importance of life history strategy for these functions has not yet been thoroughly studied. This study explored the soil-fungi relationship of two wild edible fungi, the ectomycorrhizal *Laccaria laccata* and the saprotroph *Volvopluteus gloiocephalus*. Fruiting bodies and surrounding soils in a mine-spill contaminated area were analysed. Isotope analyses revealed *Laccaria laccata* fruiting bodies were ^15^N-enriched when compared to *Volvopluteus gloiocephalus*, likely due to the transfer of ^15^N-depleted compounds to their host plant. Moreover, *Laccaria laccata* fruiting bodies δ^13^C values were closer to host plant values than surrounding soil, while *Volvopluteus gloiocephalus* matched the δ^13^C composition to that of the soil. Fungal species presented high bioaccumulation and concentrations of Cd and Cu in their fruiting bodies. Human consumption of these fruiting bodies may represent a toxicological risk due to their elevated Cd concentrations.

## Introduction

Fungi play a key role in the functioning of terrestrial ecosystems due to their diverse capabilities and relevance in nutrient cycling. Fungal functionality is directly related to their wide life history strategies, being classified as saprobes, parasites, pathogens and/or symbionts. Saprobes (or saprotrophs) are free-living fungi that obtain nutrients mostly from decomposing soil organic matter and, due to this ability, are considered as the most important decomposers in terrestrial ecosystems. Symbiotic mycorrhizal fungi also play a key role in nutrient cycling^1,2^. Mycorrhizal fungi are able to take up nutrients and water, and transfer them to the host plant, in exchange for photosynthates^3,4^. Ectomycorrhizal fungi may also mobilise soil trace elements enhancing their transfer from soil to plant and/or fungal tissues^5^. Saprotrophic fungi dominate the litter layer in soils and acquire C and nutrients in the form of organic substances by enzymatic decomposition^6^. Symbiotic ectomycorrhizal fungi are normally found in deeper soil layers (well-degraded litter and humus) with a high nutrient content but low C content, as they are not C-limited^7,8^. Ectomycorrhizal fungi are highly relevant in forests, including Mediterranean ones, as they associate with numerous tree and shrub species^9^. Previous studies found independent roles between ectomycorrhizal and saprotrophic fungi, with ectomycorrhizal fungi being able to degrade recalcitrant N-rich compounds, while saprotrophic fungi being able to degrade labile C-rich biopolymers^10^. Although ectomycorrhizal fungi have a lower ability to decompose organic matter than saprobes, they can obtain resources along the biotrophy–saprotrophy continuum^11,12^. For example, limited supply of plant photosynthate may increase enzyme production for obtaining labile carbohydrates from soil organic matter by ectomycorrizal fungi^9,13^. Ecological consequences may arise depending on the situation of the fungus within the continuum as, for example, ectomycorrhizal fungi fructification may fail if separated from plant roots^11,14^. Therefore, the spectrum of life history strategies implies that fungi differ in their roles and in their effects on ecosystem functioning.

Different mechanisms of resource acquisition, loss and cycling in fungi can be inferred by studying stable isotopes in fungal fruiting bodies (mushrooms)^15–17^. Previous studies found that ectomycorrhizal fungal sporocarps present higher δ^15^N values and lower δ^13^C values than saprotrophic ones^1,17–19^. Ectomycorrhizal fungi compete for N against saprotrophic fungi and bacteria, and they discriminate strongly against ^15^N^20^. Ectomycorrhizal fungi are known for their N isotope fractionation in the plant-soil system, as fungi retain ^15^N-enriched N while ^15^N-depleted N is transferred to the host plant. Therefore, analysing and comparing δ^13^C and δ^15^N values in fruiting bodies and underlying soils could help us to understand differences between saprotrophic and mycorrhizal strategies for resource acquisition^1^.

Fungi have been extensively studied in trace element contaminated areas as they are able to develop mechanisms to tolerate high trace element concentrations (see Bellion *et al*. 2006 for ectomycorrhizal fungi^21^). Fungi may reduce the entry of trace element concentrations into cells by a range of extracellular mechanisms, such as chelation by excreted ligands, cell-wall binding and enhanced efflux. Despite this, some amount of trace elements may still enter cells, but their toxicity can be reduced by other intracellular mechanisms, such as chelation by peptides and subcellular compartmentation^22^. In basidiomycetes, the mechanisms of response to trace element contamination are diverse and species dependent^22,23^. For example, the response of *Agaricus* to copper (Cu) is to produce metallothionein, while the response to cadmium (Cd) is to induce the production of mycophosphatin^22^.

Due to these protective mechanisms of fungi, many studies have focused on analysing the accumulation of trace elements into aboveground fungal biomass, as human consumption of edible mushrooms causes concern. In some trace element contaminated soils, a high accumulation of trace elements has been found in edible mushrooms growing there. In a silver-mining area of Czech Republic, high concentrations of trace elements were found in edible mushrooms: 149 mg kg^−1^ dry matter of Cd in *Agaricus silvaticus*, 12.9 mg kg^−1^ dry matter of mercury (Hg) in *Lepista nuda* and 16.2 mg kg^−1^ dry matter of lead (Pb) in *Lycoperdon perlatum*^24^. In another study, in a smelter area of Austria, the collected mushrooms had elevated concentrations of zinc (Zn) (up to 777 mg kg^−1^) and Cd (up to 127 mg kg^−1^)^25^.

Besides the numerous studies published, the relationships between trace element concentrations in soils and in fungal fruiting bodies are not always consistent and remain unclear. Some studies in the literature have found that fungal species may behave as bioindicators of trace elements, as concentrations in fungal fruiting bodies and their substrates correlated^26–28^. On the other hand, separate studies reported that elemental concentrations in fungi and soil did not correlate; they could have the ability to exclude trace elements or, at the contrary, to accumulate them into their biomass to levels well above (but not necessarily correlated to) the corresponding concentrations in soil^25,29^. This controversy is also affected by the soil extraction method selected to determine trace element concentration^30^. Most of the fungal studies used soil total fractions (including residual unavailable soil fractions) instead of readily or potentially available fractions^31–33^. However, the use of soil “available” fractions to compare with sporocarp concentrations is recommended^25,34^.

In order to improve our understanding of the soil-fungi relationship, the selection and analysis of the multiple factors that may explain the mushroom trace element accumulation patterns is required. First, in multi-element contaminated substrates, interactions among trace elements in soil and fungal biomass may further complicate our ability to foresee bioaccumulation patterns. In conditions of high concentrations of soil trace elements there may be a competitive interaction between some elements, for example between Cd and Zn. Some ectomycorrhizal species have a preferential uptake of Zn (essential element), through a competitive inhibition of Cd uptake (toxic element)^35,36^. Second, there are many environmental factors related to fungal accumulation of trace elements^37^. Soil variables, such as soil organic matter, pH and iron (Fe), manganese (Mn) and aluminium (Al) oxides and oxyhydroxides (among others) may explain fungal trace element accumulation, as they have a profound influence on trace element availability in the soil solution. Third, trophic differences among saprotrophs and ectomycorrhizal fungi can be related with variations in trace elements concentrations in both fruiting bodies and underlying soils, due to their different roles in soil organic matter decomposition and C and nutrient cycles^10^. Therefore, the concentration of trace elements in the soil is not the only determinant on their accumulation pattern in fungal fruiting bodies, as the soil-fungi relationship is very complex. The Bioconcentration Factor (BCF) (concentration in sporocarp divided by concentration in soil) is commonly calculated to elucidate this intricate translocation of trace elements from surrounding soil to fruiting bodies^37,38^. This BCF is therefore a very useful ratio which allows us to understand how different fungal species, fructifying in similar environmental conditions, may differ in trace element uptake and accumulation.

This study is of interest within the overall context of soil remediation processes and arises from the necessity of monitoring trace element concentrations in edible mushrooms growing in a contaminated site, as well as to elucidate the differential response of saprotrophic and ectomycorrhizal fungi to soil contamination by trace elements. We analysed the dynamics of nutrients and trace elements in two wild edible fungi species: *Laccaria laccata* (Scop.) Cooke, which was associated with stone pine and grey-leaved cistus by ectomycorrhizal symbiosis in the study area, and *Volvopluteus gloiocephalus* (DC.) Vizzini, Contu & Justo, saprotrophic fungi, fructifying in grassland soils. In a trace element contaminated and remediated ecosystem, we selected these fungal species as both were edible and fructified in the study area in the same period; in addition, they have contrasting life history strategy.

The specific objectives of this study were: (1) Determining fungal nutritional sources using C and N isotope composition; (2) Determining the accumulation capacity of trace elements in fungal fruiting bodies and the relations with soil properties; (3) Evaluating the potential toxicological risk of fruiting bodies (mushrooms) for human consumption.

## Results

### Soil properties

Soil properties in the study area were relatively homogenous for total C and N, available P, total Mg and some trace elements (Tables 1 and 2). However, there were significant differences between soils underneath fruiting bodies of ectomycorrhizal *Laccaria laccata* and saprotrophic *Volvopluteus gloiocephalus* fungi for other soil variables. Despite the fact that soil pH was acidic in the study area, *Laccaria laccata* underlying soils had lower pH (mean difference of 1.47 units) than *Volvopluteus gloiocephalus* soils (Table 1). Available K and total Ca were significantly different in the soils underneath both species, with higher concentrations in *Volvopluteus gloiocephalus* underlying soils (Table 1).

**Table 1:** Main soil chemical properties associated to *Laccaria laccata* and *Volvopluteus gloiocephalus* fungal species.

*Soil properties*	*Laccaria laccata*	*Volvopluteus gloiocephalus*	*t*	*p*
pH	4.6 ± 0.2	6.1 ± 0.2	−7.00	**<0.001**
C_total_ (%)	0.901 ± 0.152	1.09 ± 0.12	−1.00	0.330
N_total_ (%)	0.169 ± 0.015	0.195 ± 0.010	−1.49	0.157
P_available_ (mg kg^−1^)	11.5 ± 2.7	10.8 ± 0.8	1.22^*^	0.249
K_available_ (mg kg^−1^)	166 ± 16	272 ± 14	−5.07	**<0.001**
Ca_total_ (mg kg^−1^)	2127 ± 149	3469 ± 130	−6.79	**<0.001**
Mg_total_ (mg kg^−1^)	3014 ± 98	3122 ± 92	−0.81	0.428

Mean values (±SE) for N = 10. Student’s *t*-test statistic and *p* value (in bold *p* < 0.05) are shown. ^*^Data transformation for normality of the residuals.

**Table 2:** Soil trace elements and S (pseudo-total and CaCl_2_-extractable) associated with *Laccaria laccata* and *Volvopluteus gloiocephalus* fungal species.

*Soil trace elements*	*Laccaria laccata*	*Volvopluteus gloiocephalus*	*BV (P 90)*	*t*	*p*
**Pseudo-total**					
As	142 ± 7	123 ± 15	157	1.12	0.282
Cd	0.630 ± 0.062	1.08 ± 0.05		−5.59	**<0.001**
Co	8.38 ± 0.47	11.2 ± 0.4	34	−4.54	**<0.001**
Cu	174 ± 13	185 ± 8	108	−0.73	0.475
Fe	37222 ± 1187	35973 ± 1902		0.56	0.586
Mn	301 ± 18	418 ± 17		−4.66	**<0.001**
Ni	14.7 ± 0.4	14.9 ± 0.4	62	−0.34	0.737
Pb	227 ± 14	204 ± 26	117	0.77	0.456
S	2421 ± 319	1769 ± 438		−2.07^§^	0.058
Zn	193 ± 10	263 ± 11	134	−4.67	**<0.001**
**CaCl**_**2**_**-extractable**					
Cd	0.142 ± 0.019	0.042 ± 0.008		4.91	**<0.001**
Co	0.145 ± 0.038	0.019 ± 0.005		4.51^§^	**<0.001**
Cu	1.72 ± 0.70	0.253 ± 0.031		−4.68^§^	**<0.001**
Fe	4.90 ± 0.42	4.30 ± 0.87		0.62	0.544
Mn	15.2 ± 1.9	4.52 ± 0.88		5.19	**<0.001**
Ni	0.354 ± 0.036	0.103 ± 0.015		6.45	**<0.001**
Pb	0.082 ± 0.015	0.083 ± 0.021		−0.06	0.957
S	135 ± 75	74.5 ± 53.2		62^◊^	0.182
Zn	14.8 ± 1.9	3.12 ± 0.91		5.66	**<0.001**

Mean values (±SE) in mg kg^−1^ for N = 10. Student’s *t*-test statistic and *p* value (in bold *p* < 0.05). Background values (BV) at percentile 90 (P 90) in South Portuguese Zone soils at depth 0–20 cm in mg kg^−1^ are indicated^83^. ^§^Data transformation for normality of the residuals. ^◊^Non-parametric Mann-Whitney test (U statistic).

Soil pseudo-total arsenic (As), Cu, Fe, nickel (Ni), Pb and sulphur (S) were relatively homogeneous in the study area. However, pseudo-total Cd, cobalt (Co), Mn and Zn differed between fungal species habitats, being significantly higher the concentrations in *Volvopluteus gloiocephalus* underlying soils (Table 2). Soil “available” (CaCl_2_-extracted) concentrations of trace elements presented a different trend than pseudo-total values. Soil underneath *Laccaria laccata* sporocarps had significantly higher CaCl_2_-extracted concentrations for almost all trace elements (Cd, Co, Cu, Mn, Ni and Zn), except for Fe, Pb and S concentrations that were similar in both soils (Table 2).

### Carbon and nitrogen isotopes in soil and fungal sporocarps

**Figure 1:**
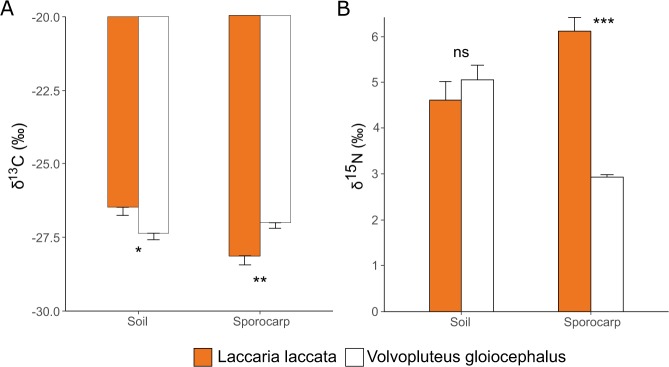
(**A**) Carbon and (**B**) Nitrogen isotope composition in soil and sporocarps of *Laccaria laccata* and *Volvopluteus gloiocephalus* fungal species. Bars represent mean values and error bars represent standard error for N = 10. *P* values resulting from Student’s *t*-test are indicated (ns: non-significant; *p* < 0.05*; *p* < 0.01**; *p* < 0.001***).

Soil C content was similar for both fungal species, however C isotope composition of these soils was different; *Laccaria laccata* surrounding soils were ^13^C-enriched compared to *Volvopluteus gloiocephalus* ^13^C-depleted surrounding soils (Table 1 and Fig. 1A). Ectomycorrhizal *Laccaria laccata* fungal sporocarps were ^13^C-depleted relative to saprotrophic *Volvopluteus gloiocephalus* fungi (Fig. 1A). *Laccaria laccata* fruiting bodies were ^13^C-depleted compared to their soil (t = 4.24; *p* < 0.001), whereas *Volvopluteus gloiocephalus* fruiting bodies were not significantly different to their soil (t = −1.31; *p* = 0.208) (Fig. 1A).

Soil N content was similar for both fungal species as well as their N isotope composition (Fig. 1B). In contrast to δ^13^C values, ectomycorrhizal *Laccaria laccata* sporocarps were ^15^N-enriched in comparison to saprotrophic *Volvopluteus gloiocephalus* sporocarps. Ectomycorrhizal *Laccaria laccata* sporocarps were ^15^N-enriched compared to the surrounding soil (t = −2.99; *p* = 0.009), whereas saprotrophic *Volvopluteus gloiocephalus* fungi were ^15^N-depleted (t = 6.51; *p* < 0.001) (Fig. 1B).

### Chemical composition of fungal sporocarps

**Figure 2:**
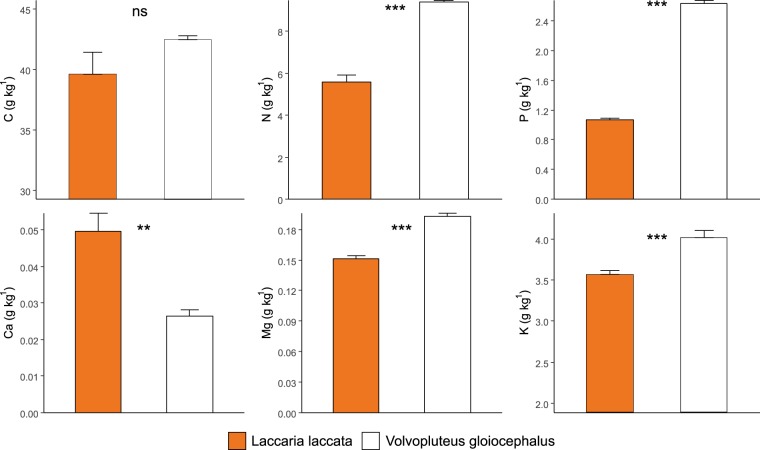
Nutrients in *Laccaria laccata* and *Volvopluteus gloiocephalus* sporocarps. Bars represent mean values and error bars represent standard error for N = 10. *P* values resulting from Student’s *t*-test are indicated (ns: non-significant; *p* < 0.01**; *p* < 0.001***).

There were differences in nutrient concentrations between the fungal species. *Volvopluteus gloiocephalus* fruiting bodies presented significantly higher contents of N, P, K and Mg compared to *Laccaria laccata* sporocarps, whilst this tendency was the opposite for Ca, with higher concentrations in *Laccaria laccata* fruiting bodies. There was no significant difference between fungal species regarding C content (Fig. 2).

**Figure 3:**
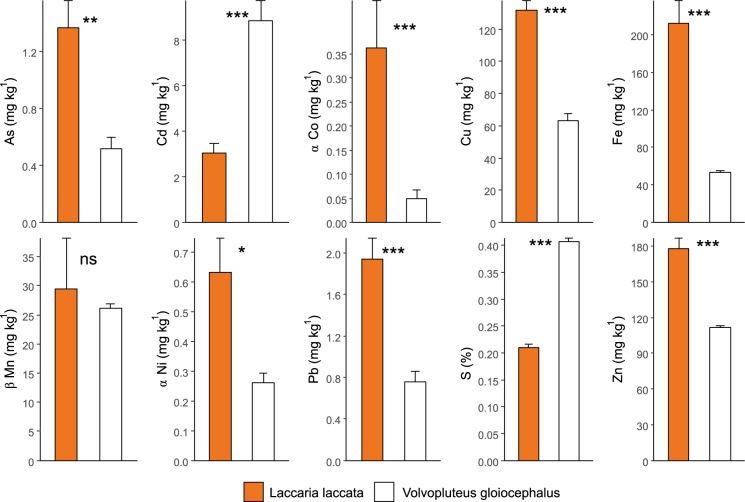
Trace elements and S in *Laccaria laccata* and *Volvopluteus gloiocephalus* sporocarps. Bars represent mean values and error bars represent standard error for N = 10. Student’s *t*-test statistic and *p* value are indicated (ns: non-significant; *p* < 0.05*; *p* < 0.01**; *p* < 0.001***). ^α^ Data transformation for normality of the residuals. ^β^ Non-parametric Mann-Whitney test (U statistic).

The two fungal species had different patterns of accumulation for most of the trace elements and S (Fig. 3), with higher concentrations of As, Co, Cu, Fe, Ni, Pb and Zn in *Laccaria laccata* fruiting bodies, and of Cd and S in *Volvopluteus gloiocephalus* fruiting bodies. No significant differences were found for Mn among fungal sporocarps of these species.

### Bioconcentration factors of trace elements

Cadmium BCF was high in both fungal species (means of 5.09–8.49; Table 3). Mean BCF values of Co, Cu and Zn were close to one (and maximum values exceeded the unity) in the mycorrhizal *Laccaria laccata*, but not in the saprotrophic *Volvopluteus gloiocephalus*. The rest of the trace elements (As, Fe, Mn, Ni, Pb) and S had low BCF values for both species.

**Table 3:** Bioconcentration factor (BCF) of pseudo-total trace elements and S in *Laccaria laccata* (*L*) and *Volvopluteus gloiocephalus (V)*. Bold values indicate BCF > 1.

BCF	Species	Mean	SD	Min	Max
As	*L*	0.0096	0.0040	0.002	0.015
	*V*	0.0044	0.0021	0.002	0.009
Cd	*L*	**5.09**	**2.87**	**2.75**	**12.58**
	*V*	**8.49**	**3.34**	**4.98**	**13.66**
Co	*L*	0.626	0.602	0.204	**2.286**
	*V*	0.034	0.042	0.001	0.138
Cu	*L*	0.795	0.217	0.462	**1.186**
	*V*	0.351	0.114	0.243	0.634
Fe	*L*	0.0057	0.0018	0.031	0.0079
	*V*	0.0015	0.0003	0.001	0.0021
Mn	*L*	0.107	0.119	0.049	0.442
	*V*	0.064	0.011	0.040	0.077
Ni	*L*	0.044	0.027	0.018	0.100
	*V*	0.018	0.007	0.006	0.028
Pb	*L*	0.0086	0.0021	0.006	0.012
	*V*	0.0040	0.0027	0.002	0.011
S	*L*	0.0001	<0.0001	0.0001	0.0002
	*V*	0.0003	0.0002	0.0001	0.0006
Zn	*L*	0.942	0.173	0.715	**1.296**
	*V*	0.432	0.066	0.351	0.561

**Figure 4:**
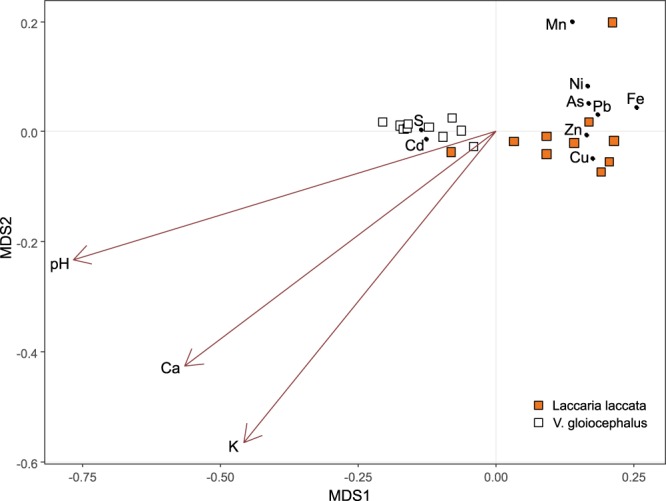
Nonmetric multidimensional scaling (NMDS) ordination of trace element bioconcentration factors (BCF) of *Laccaria laccata* (orange squares) and *Volvopluteus gloiocephalus* (white squares) fungal species. Black dots represent BCF for each trace element and S. Brown arrows represent soil variables with a significant correlation with BCF (*p* = 0.001 for pH and K, *p* = 0.003 for Ca).

According to the NMDS ordination, the soil variables that best explained BCF variability were pH, total Ca and available K (Fig. 4). *Volvopluteus gloiocephalus* sporocarps fructified in soils with higher pH and higher Ca and K concentrations compared to *Laccaria laccata* sporocarps. Two defined clusters separated the fungal species with a higher BCF of Cd and S in *Volvopluteus gloiocephalus*, while *Laccaria laccata* sporocarps presented higher BCF for the rest of the elements (Fig. 4).

The transfer ratio of trace elements from soil (CaCl_2_-extracted fraction) to fungal sporocarps was very high for Cd and Cu, moderate for Zn and Fe, low for Co, Mn and Ni, and negligible (<1) for S (Fig. S1). The transfer ratios from soil to fungus were higher in *Volvopluteus gloiocephalus*, in particular for Cd (x12), Zn (x6) and Mn (x5), with the exception of Fe which was higher (x2) in *Laccaria laccata*.

In general, Pearson´s correlation coefficients between trace element concentrations in fungal sporocarps and their underlying soils (CaCl_2_-extracted values) were low (Table S1). *Laccaria laccata* had a significant correlation (*p* < 0.05) with Co, and marginally significant correlations (*p* < 0.10) with As and Mn, and *Volvopluteus gloiocephalus* with Fe.

### Toxicity risk

Calculations of the minimum daily intake of the studied fruiting bodies to reach maximum tolerable intake limits for toxicity in humans provided weights over 1 kg of fresh weight of both species for As, Cu, Fe, Pb and Zn. However, the consumption of 0.069 kg fresh weight of *Volvopluteus gloiocephalus* or 0.132 kg fresh weight of *Laccaria laccata* would be enough to reach the daily limit for Cd (Table S2). (All the calculations were based on a person of 70 kg bodyweight).

## Discussion

Soil microorganisms play a key role in nutrient cycling, plant symbioses, organic matter decomposition, and other ecosystem processes^39^. Trace element contaminated soils are of concern due to their toxic effects on soil microbes^40^. In these soils, fungal communities provide many ecosystem services of regulation; in general, improving soil and water quality, nutrient cycling, soil fertility and carbon sequestration^41^. In particular, the “mycoremediation” potential to stabilise trace elements in fungal tissues can be considered as another ecosystem service. Thus, fungal mycelium secretes extracellular enzymes and acids to break down organic contaminants and has a high metal-binding capacity playing a promising role in remediation of trace elements^42^. However, from fungal mycelium, trace elements could be channelled to fruiting bodies implying a nutritional potential hazard due to mushroom consumption^42^. Fruiting bodies (mushrooms) of more than 1100 fungal species are used worldwide for food and medicines, thus delivering provision and cultural services^43^. With exception of poisonous species or edible fungi with high concentration of potentially toxic trace elements, which are a hazard for human health, and then represent a cultural “disservice”^41^.

The large-scale phytoremediation plan within the contaminated area considered in this study included planting of native trees and shrubs^44^. With time, planted trees modified the properties of underlying soil, for example, pine trees induced soil acidification and, consequently, higher concentrations of soluble fractions of trace elements^44,45^. Trees also influenced surrounding soil microbial activities, compared with adjacent treeless patches^46^. In this heterogeneous landscape mosaic the two studied fungal species fructified in contrasting habitats and soils: *Laccaria laccata* was associated with their host pine trees while saprotrophic *Volvopluteus gloiocephalus* was abundant in the grasslands without trees. Soils surrounding *Volvopluteus gloiocephalus* sporocarps presented higher pseudo-total concentrations of some elements (Cd, Co, Mn and Zn) than *Laccaria laccata* soils (under pine trees). These differences may be caused by the acidification process which increases losses of trace elements from soil due to enhanced leaching and take up by vegetation and fauna^47^. However, elements with low mobility, such as As and Pb, had similar concentrations between soil types. The opposite pattern was found for CaCl_2_-extracted trace element values; most of the trace elements were found in higher concentrations in soils surrounding *Laccaria laccata* sporocarps under pine trees probably due to the pine-induced soil acidification and subsequent trace element mobilisation, as already mentioned.

The differences in habitat and in life history strategy influence the mineral nutrition of both fungal species. *Laccaria laccata* sporocarps were found in soils close to their hosts *Pinus pinea* and *Cistus albidus*, with which they develop symbiotic relationships. The lower nutrient concentration of the mycorrhizal sporocarps, compared with those of *Volvopluteus gloiocephalus*, could be explained by the symbiotic association as pine and cistus may receive part of the nutrients taken up by this fungi, such as N and P^10,19^. In contrast, the saprotrophic *Volvopluteus gloiocephalus*, found in grassland areas dominated by herbaceous species, completely relies on soil sources for nutrition without loss by transfer to other organisms. The nutritional quality of both fungal sporocarps was within the usual content range for wild growing mushrooms, except for Mg that was lower in both species^48^.

Contrasting life history strategy of the studied fungal species influences C and N cycles. Despite similar soil C content, the observed differences in soil δ^13^C values may reflect a different isotope composition or different turnover rates, and the preferential use of ^12^C compared to ^13^C in biological processes^49^. Carbon isotope composition in *Volvopluteus gloiocephalus* fruiting bodies matched the ratios of the C soil source, according to its saprotrophic nature as litter decay fungi. In contrast, *Laccaria laccata* fruiting bodies were ^13^C-depleted (−28.14‰) compared to soil (−26.49‰), reflecting the isotope composition of its host photosynthate, which could be the main C source^1,50^. The differences in N isotope composition between both fungal species were even more conspicuous, where ectomycorrhizal *Laccaria laccata* fruiting bodies were ^15^N-enriched in comparison to the surrounding soil; potentially because of the transfer of ^15^N-depleted compounds to the symbiotic plants^1,18,51–55^. Whereas, saprotrophic *Volvopluteus gloiocephalus* fruiting bodies had much lower ^15^N than *Laccaria laccata*, and were ^15^N-depleted in comparison to the soil, despite having similar N isotope composition in both soil types.

A previous study in Sweden found fungi were able to take up and accumulate trace elements such as Cd, Cu and Zn in both sporocarps and mycelium (BCF from 1.9 to 8.8) in respect of bulk soil concentrations^56^. In this study, we found that the fungal uptake of trace elements is dependent on both the chemical element and the fungal species^5^. Low selectivity of fungal transporters for essential elements, such as Ca and Zn, favoured the transport of toxic ions with similar chemical properties, such as Cd^22^. In the study area, both *Volvopluteus gloiocephalus* and *Laccaria laccata* sporocarps mainly bioconcentrated Cd. Previous studies in the same area had found that roots of seven tree species also concentrated Cd (BCF > 1), making them suitable for the phytostabilisation of this potentially toxic element^44^. Although Cd was accumulated in fruiting bodies of both fungal species, the patterns and relations with soil differed; *Volvopluteus gloiocephalus* sporocarps accumulated much higher levels of Cd than *Laccaria laccata*. However, accumulation of Ca and Zn were opposite, with higher concentrations in *Laccaria laccata* sporocarps. The correlation between Cd levels in fruiting bodies and soils was positive for the saprotrophic species but negative for the ectomycorrhizal one; a possible explanation is that Cd-Zn-Ca competition influence elements accumulation in the studied sporocarps^57^. A higher Ca and Zn content was registered in *Laccaria laccata* sporocarps, besides *Volvopluteus gloiocephalus* surrounding soils recorded a higher Ca and Zn concentrations. Therefore, a competition among Cd-Zn-Ca could be responsible for the preference of translocation of Ca and Zn over Cd in *Laccaria laccata* fruiting bodies. On the other hand, the presence of Cd could induce the production of intracellular binding compounds such as metallothioneins, cadystin, and phytochelatins, and cellular compartmentalization in this species^22,58,59^. Besides the antagonism or interaction between chemical elements, the bioconcentration of trace elements in fungal fruiting bodies is influenced by soil properties^60^. In this study, soil pH, Ca and K were relevant variables explaining the BCF of trace elements in fruiting bodies, and these variables were positively correlated among them (*p* < 0.001). In acidic soils, the release of most trace elements increases, being more available in the soil solution and, therefore, more accessible to microorganisms^61,62^. In comparison with other studies, As concentrations in *Laccaria laccata* sporocarps are broad, depending on the location and soil conditions, with a range from 0.66 to 147 mg kg^−1^ in caps and stipes^63^.

The CaCl_2_-extracted fraction for most of the trace elements in the soil usually represents a small proportion of their total concentration (Table 2). Therefore, the transfer ratios of trace elements from soil (CaCl_2_-extracted pool) to fungal sporocarps are functionally more realistic than BCF values (calculated using pseudo-total soil concentrations) and worthwhile to consider. Thus, besides the important Cd accumulation already discussed above, we have detected a high transfer of Cu from soil to fungus (over 200 times). Other trace elements with remarkable soil to fungus transfer were Zn (78 times more in *Volvopluteus gloiocephalus*) and Fe (48 times more in *Laccaria laccata*). If we assume that sporocarps are reflecting mycelium chemical composition, we can infer that both fungal species are contributing potentially to the “mycostabilisation” of Cd, Cu, Zn and Fe in these contaminated and remediated soils^37,42^.

The well-known capacity of mushrooms to accumulate trace elements could make them useful organisms to bioindicate soil contamination^64^. However, in this study, trace element concentrations in fruiting bodies were not significantly correlated to soil CaCl_2_-extracted concentrations, with the exception of Co in *Laccaria laccata*. Many studies have also found that fruiting bodies are not correlated with soil contamination by trace elements; therefore concluding that they are not good bioindicators of soil trace element contamination^32,65–67^. On the contrary, there are authors supporting the use of fungal sporocarps as bioindicators^26–28^. In our study site, the concentration of trace elements in leaves of poplar trees and in soil were correlated for Cd, Cu, Mn and Zn^68^. The potential utility as bioindicator would then depend on the fungal species, the particular trace element, and the environmental conditions.

From the food quality perspective, the usually high accumulation of potentially toxic elements in fungal fruiting bodies is a relevant ecosystem disservice. Trace elements concentrations in fruiting bodies were similar to those measured in edible mushrooms from unpolluted areas^67^, except for the increased mean concentrations of Cd in *Volvopluteus gloiocephalus* (and maximum in *Laccaria laccata*) and mean Cu and maximum Fe, Mn and Zn concentrations in *Laccaria laccata* sporocarps (Table S3).

Food regulation to avoid toxicity risk includes concentration limits for several trace elements in wild edible mushrooms. Thus, the European Union regulation established a concentration limit of 0.3 mg Pb kg^−1^ fresh weight and 0.2 mg Cd kg^−1^ fresh weight for cultivated fungal species (*Agaricus bisporus*, *Pleurotus ostreatus* and *Lentinula edodes*). For other fungal species, this limit was established at 1.00 mg Cd kg^−1^ fresh weight and no regulation limit was established in other species for Pb^69,70^. The maximum Cd concentration registered in *Volvopluteus gloiocephalus* sporocarps was 0.84 mg Cd kg^−1^ fresh weight, which is well above the European Union regulation limit for cultivated mushrooms, although still below the limit for wild species. The maximum Pb concentration in our sporocarps was 0.25 mg Pb kg^−1^ fresh weight, which is also below the limit (for cultivated species only). Therefore, according to European Union regulation, neither Cd nor Pb concentrations reached these maximum levels (Table S3; dry weight).

The World Health Organization^71^ evaluated certain trace element contaminants in food to estimate the tolerable intake limits (Table S2; PMTDI). We calculated the minimum weight to reach tolerable limits, and Cd presented the lowest weight (Table S2). A daily intake of 0.069 kg fresh weight of *Volvopluteus gloiocephalus* or 0.132 kg fresh weight of *Laccaria laccata* (for a person with 70 kg bodyweight) would reach the maximal tolerable Cd daily limit, without taking into account other Cd daily sources. The consumption of these fungi could represent a toxicological risk due to elevated Cd content, especially *Volvopluteus gloiocephalus* species, as this element is of high toxicological importance for humans^67,72^. *Laccaria laccata* consumption would entail a higher toxicological risk than *Volvopluteus gloiocephalus* for the rest of trace elements (As, Cu, Fe, Pb and Zn), however the daily intake to reach toxicity (over 1 kg fresh weight) represents an unrealistically high level of consumption. In any case, for a complete evaluation of actual potential toxicity to humans the effect of mushroom processing during cooking on its metal concentrations should also be taken into account.

## Conclusions

After a long-term restoration process to mitigate the effects of a mine-spill in SW Spain, *Laccaria laccata* and *Volvopluteus gloiocephalus* sporocarps and surrounding soil analyses demonstrated trace element persistence in the study area. Some soil trace element concentrations were above background values and soil acidification seemed to be responsible for the higher trace element concentrations in *Laccaria laccata* fruiting bodies. Analyses of δ^15^N and δ^13^C in fruiting bodies and surrounding soils allowed us to differentiate the saprotrophic *Volvopluteus gloiocephalus* fungi from the ectomycorrhizal *Laccaria laccata* fungi, proving this technique as adequate to differentiate fungal life history and to contribute to a better knowledge of the soil-fungi relations.

Bioconcentration factors demonstrated that both fungal species accumulated Cd, and that Cu was transferred from the soil “available” (CaCl_2_-extracted) pool to fungal sporocarps. Human consumption of *Laccaria laccata* and/ or *Volvopluteus gloiocephalus* sporocarps collected in the area may represent a toxicological risk due to the elevated concentrations of Cd. Therefore, strict control is required in the area to avoid human consumption of these fruiting bodies. Due to the ability of these species to accumulate some metals, monitoring on trace element concentrations in these fungi is thus highly recommended, even if mobility of metals in soils is assumed to be low.

## Materials and methods

### Study area

The study was conducted in the Guadiamar Green Corridor (Seville, SW Spain; 37°23.165′N, 6°13.668′W) which is a public area managed by the Spanish Regional Government. The study area is located 12 km downstream from the Aznalcóllar tailings dam failure, which affected about 4000 ha and a 60 km stretch along the Guadiamar River^73,74^. Aznalcóllar mine is located in the Iberian Pyrite Belt formed by a large concentration of polymetallic massive sulphides with high grade on silver (Ag), Pb and Zn^74^. The mine-spill covered these soils with 6 hm^3^ of acidic waters and sludge loaded with a mixture of trace elements, mainly As, Cd, Cu, Pb, thallium (Tl) and Zn^75^. Since the accident (in 1998) until 2001, a remediation plan was put in place which consisted in sludge and top soil removal by heavy machinery and application of amendments (sugar lime, clay and organic matter) to immobilise trace elements. Subsequently, afforestation of the area with native trees and shrubs was carried out^44^. Differences in soil type and remediation outcome created a heterogeneous Corridor, and 15 years after the remediation of the area some patches still presented high soil acidification and trace element content^76^. The study area is one of these degraded areas with high toxicity risks due to the naturally sandy and acidic soil, with a limited capacity of soils to buffer against acid drainage caused by the remnants of mining residues.

### Study fungal species

**Figure 5:**
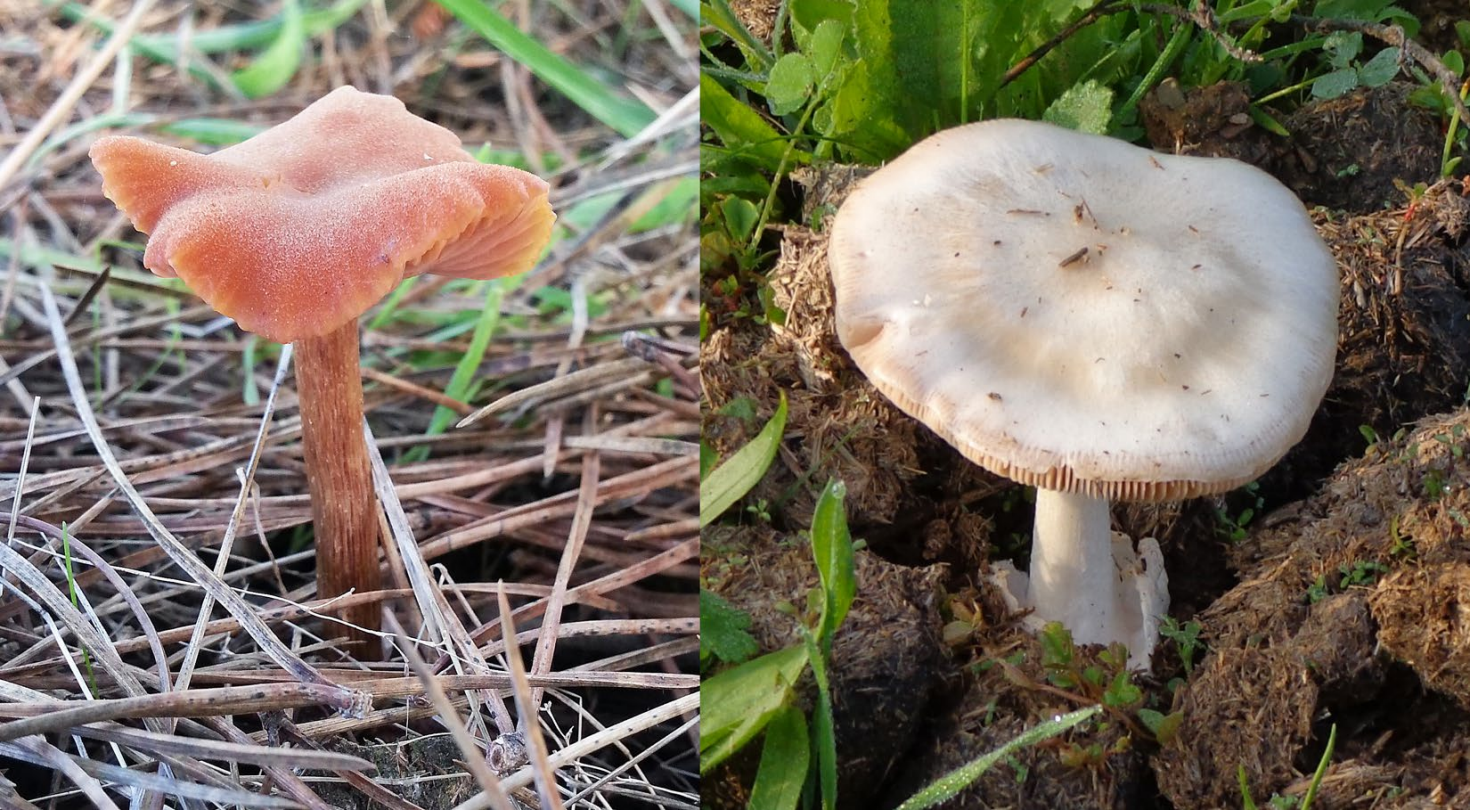
Fruiting bodies of the ectomycorrhizal *Laccaria laccata* (left) over needles of *Pinus pinea*, and of the saprophytic *Volvopluteus gloiocephalus* (right) over open grassland. Photos were taken in December 2016 in the Guadiamar Green Corridor, SW Spain (authors: T. Marañón and C. M. Navarro-Fernández).

The two selected species in this study were *Laccaria laccata*, which form ectomycorrhizae, and *Volvopluteus gloiocephalus*, which is a saprobe (Fig. 5). Both species belong to the Basidiomycota division and Agaricales order, but are classified in different families. *Laccaria laccata* belongs to the Hydnangiaceae family and *Volvopluteus gloiocephalus* to the Pluteaceae family. *Laccaria laccata* is distributed broadly in the Northern Hemisphere and has small, red-orange fruiting bodies. This fungal species has a wide ectomycorrhizal host potential, a low specificity and it is characteristic of young stands of trees^77^. This species has been identified as component of the fungal ectomycorrhizal communities associated with *Quercus ilex* in the study area^5,78^. *Volvopluteus gloiocephalus* is distributed in Europe and North America and has bigger fruiting bodies than *Laccaria laccata* with white or grey-brown colours^79^.

The specimens of fruiting bodies collected for this work were registered in the University of Seville Herbarium to the codes SEV-C 51 for *Laccaria laccata* and SEV-C 50 for *Volvopluteus gloiocephalus*. Molecular analyses were carried out to confirm the species morphological determination and to identify potential ecotypes. Sequences are available in GenBank (https://www.ncbi.nlm.nih.gov/genbank/) under accession numbers MK616245 - MK616246 for *Laccaria laccata* and MK616344 - MK616347 for *Volvopluteus gloiocephalus*.

### Sampling design

In December 2016, a field sampling was conducted in order to evaluate the wild growing edible fungal fruiting bodies that fructified in the remediated soils of this area. Fruiting bodies were sampled in the locations where they fructified, naturally occurring in separated habitats. *Laccaria laccata* sporocarps were found close to the ectomycorrhizal tree, *Pinus pinea*, and the shrub, *Cistus albidus*. In contrast, *Volvopluteus gloiocephalus* sporocarps were found in adjacent treeless areas covered by different herbaceous species. Therefore, sampling locations were dependent on the distribution of these habitats in the study area (Fig. S2). For each species, ten points and at least three sporocarps per point were sampled. Soil surrounding each sporocarp was taken (top 0–5 cm depth) with an auger from at least five locations within each point to make a composite sample per point. Sporocarps and soils were kept refrigerated until being processed the day after sampling.

### Soil analyses

Soil samples (top 0–5 cm depth; 100 g fresh weight) were air-dried and sieved to <2 mm for chemical analysis, where soil pH was measured in a 1:2.5 soil-water suspension after 30 min of shaking. Bray 1 method was selected to determine soil P in these acidic soils^80^. Available K was extracted with 1 M ammonium acetate and determined by multiparametric Bran-Luebbe autoanalyser. Pseudo-total concentrations of trace elements (As, Cd, Co, Cu, Fe, Mn, Ni, Pb and Zn), S, Ca and Mg were extracted by digestion of soils, ground to <60 µm, with *aqua regia* (1:3 v/v conc. HNO_3_/ HCl) in a Digiprep MS block digester (SPS Science) for 2 h at 110 °C. Trace elements (As, Cd, Co, Cu, Fe, Mn, Ni, Pb and Zn) and S were extracted from <2 mm sieved soils with a 0.01 M CaCl_2_ solution to estimate their “bioavailability”^81^. Extracts after digestion with *aqua regia* or CaCl_2_, were determined by inductively coupled plasma optical emission spectrophotometry (ICP-OES) with Varian ICP 720-ES. The quality of the analyses was assessed using the reference sample ERM-CC141 (loam soil) and recoveries from 93.8% to 100.4% were obtained. Arsenic extracted by CaCl_2_ presented concentrations below detections limits (0.005 mg kg-1), and its “availability” is underestimated by this method.

### Chemical composition of fungal fruiting bodies

Fruiting bodies were dry cleaned with a brush and fresh weighed, with some biomass kept at −80 °C for further species identification by molecular analysis. Sporocarps were dried at 70 °C for 48 h and stored until analysis. The whole fruiting body (cap, hymenophore and stalk) was analysed as one single sample, as all the parts are usually ingested when consumed by humans. Fruiting bodies macronutrients (Ca, K, Mg, S and P) and trace elements were extracted by digestion with HNO_3_ in a Digiprep MS block digester (SPS Science) for 2 h at 110 °C and determined by ICP-OES with Varian ICP 720-ES. Arsenic was determined by ICP-OES with Ion Exchange Hydride Generation (IE-HG-ICP-OES) methodology^82^. The quality of the analysis was assessed using the reference sample ID 124 Type Lucerne (*Medicago sativum*) (WEPAL; IPE) for As, with recoveries of 95.9%, and the reference sample INCT-OBTL-5 (tobacco leaves) for the rest of the trace elements, with recoveries from 79.9% to 112.2%.

### Isotopic analysis

Total C and N and their isotope composition (δ^13^C and δ^15^N, respectively) in soil and sporocarps were determined by elemental analysis and isotope ratio mass spectrometry (EA/IRMS) system by means of Flash HT Plus elemental analyser coupled to a Delta-V Advantage isotope ratio mass spectrometer via a CONFLO IV interface (Thermo Fisher Scientific, Bremen, DE). The analytical measurement errors were ±0.1‰ for δ^13^C and ±0.2‰ for δ^15^N. Total C was organic C due to the absence of carbonates in the study area^76^.

### Data analysis

Mean and standard error (SE) of datasets from *Laccaria laccata* and *Volvopluteus gloiocephalus* sporocarps and respective soils were calculated. The chemical composition of soils and sporocarps of the studied species were compared through a parametric Student’s t-tests. Normality assumptions were verified with Lilliefors-corrected Kolmogorov-Smirnov test for normality and Levene test for homogeneity of variance. If the assumptions were not met, the data was Box-Cox transformed. If this transformation did not meet the assumptions, a non-parametric Mann-Whitney test was subsequently performed.

To evaluate the level of contamination, the values of soil pseudo-total concentration of trace elements were compared with background values (BV) for the South Portuguese Zone^83^. The reference level (percentile 90), which is the maximum value accepted for non-contaminated soils, was used.

Bioconcentration factors of trace elements were calculated as concentration in fruiting bodies divided by pseudo-total concentration in soil underneath (both in mg kg^−1^)^37,38^. To interpret the results, a BCF > 1 suggests that fungi have some transporters (active or passive) that can carry trace elements into fungal biomass resulting in accumulation, while a BCF < 1 may indicate the existence of fungal mechanisms against take up of trace elements^30^. In addition, the ratio between trace elements accumulated in the fungal fruiting bodies and those “bioavailable” in the soil (estimated by CaCl_2_ extraction)was calculated as a functional transfer ratio^34^.

To explore relationships among sporocarps BCF and soil variables, a nonmetric multidimensional scaling (NMDS) was performed^84^. The BCF data was standardised with a Hellinger transformation and selected Euclidean distance metrics, where the number of dimensions was k = 2 with an adequate level of stress of 0.010. Soil variables effects were calculated using a permutation model (with 999 permutations) and fitted using the *envfit* function^85^. In this plot, the length of arrows represents the strength of the correlation and its direction indicates the trend of change in the variable.

To evaluate relationships of trace elements in the soil (CaCl_2_-extracted fraction) with sporocarps we performed a Pearson´s correlation test, adjusted with Benjamini-Hochberg correction^86^.

All data analyses were performed utilising the R software package v3.5.1^87^ using *ggplot2*^88^, *lawstat*^89^, *MASS*^90^, *nortest*^91^, *psych*^92^ and *vegan*^85^ packages.

### Evaluation of toxicity risk

Fruiting bodies concentrations of potentially toxic elements (Cd and Pb) in the two fungal species were compared with the regulation limits (in fresh weight) for edible mushrooms of the European Union^69,70^. The fruiting bodies concentrations of Cd and Pb were transformed from dry weight to fresh weight by dividing the limits (Cd limited to 1 mg kg^−1^ fresh weight; Pb limited to 0.3 mg kg^−1^ fresh weight) by the mean fresh weight (*Laccaria laccata* 7.13 g fresh weight; *Volvopluteus gloiocephalus* 5.77 g fresh weight) (Table S3).

Fungi minimum daily intake to reach the upper level of the tolerable intake threshold, established by the Joint FAO/WHO Expert Committee on Food Additives^93^, were calculated to those trace elements reported in that guideline: As, Cd, Cu, Fe, Pb and Zn. The guideline limits were transformed into provisional maximum tolerable daily intake (PMTDI) and the weights were expressed as kg of fresh weight for a person with 70 kg bodyweight (as proposed by the European Food Safety Authority) (Table S2).

## Supplementary information

Supplementary Information.
