# Supplementary material for: Trace elements and C and N isotope composition in two mushroom species from a mine-spill contaminated site
Source: Sci Rep. 2020 Apr 15;10:6434. doi: 10.1038/s41598-020-63194-2 (PMC7160199; doi:10.1038/s41598-020-63194-2)
Supplement: Supplementary file 1 — Supplementary Information. [file 41598_2020_63194_MOESM1_ESM.docx]

**Supplementary Material**

**Article title:** Trace elements and C and N isotope composition in two mushroom species from a mine-spill contaminated site

**Authors:** Marta Gil-Martínez*, Carmen M. Navarro-Fernández, José M. Murillo, María T. Domínguez, Teodoro Marañón

*** Correspondence:** Marta Gil-Martínez; marta.gil@irnas.csic.es

**Figure Supplementary 1.** Transfer ratio (in logarithmic scale) of eight trace elements from soil (CaCl_2_-extractable pool) to fungal sporocarps for the two study species: *Laccaria laccata* (orange bars) and *Volvopluteus goiocephalus* (white bars).

**Figure Supplementary 2.** Location map of the studied area within the Guadiamar Green Corridor (SW Spain) with sampled points of *Laccaria laccata* (orange squares) and *Volvopluteus gloiocephalus* (white squares). Map image form Google Earth, version 7.3.2.5776 (earth.google.com/web/).


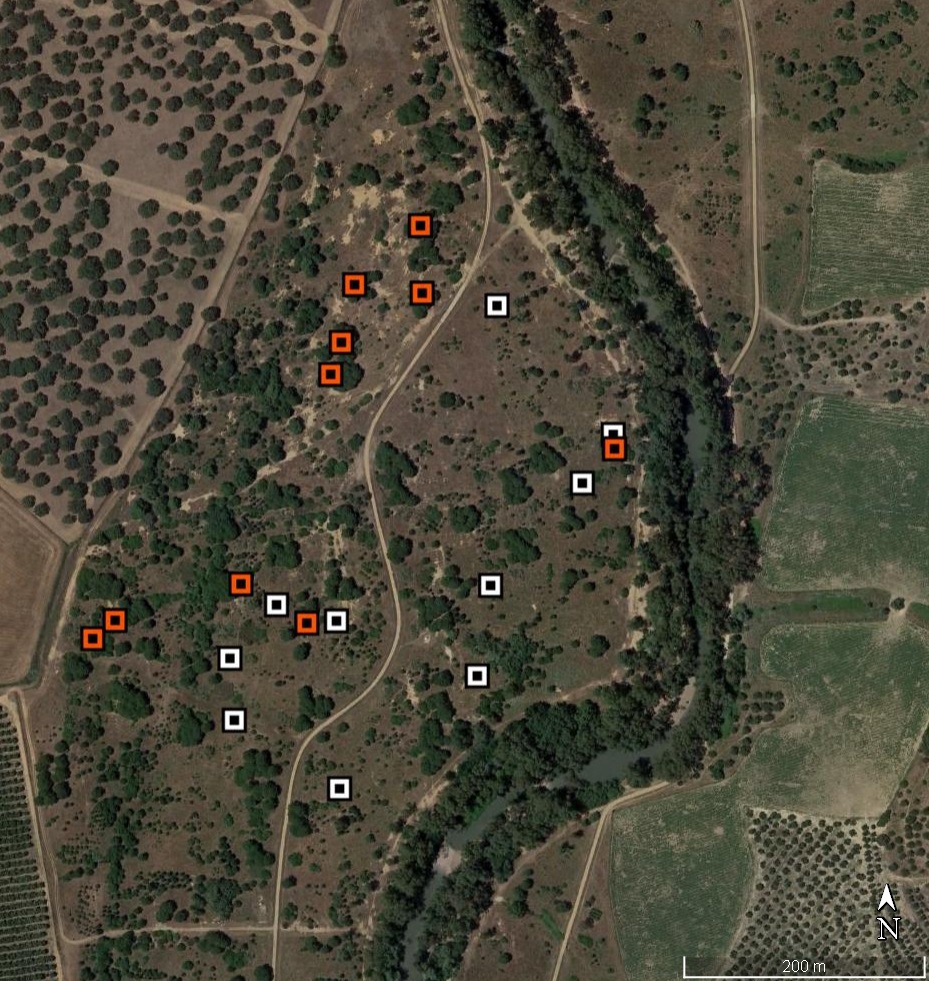


© 2019 Google


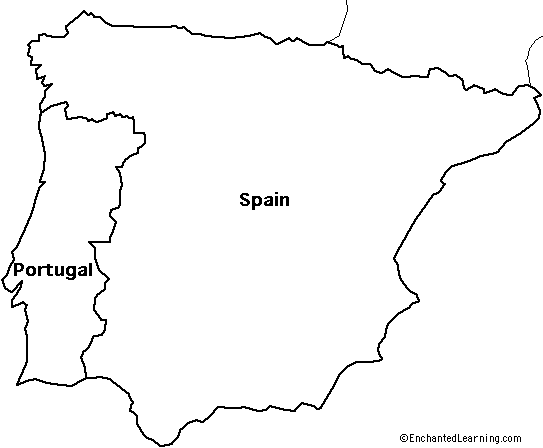


**Table Supplementary 1**. Correlations between trace elements in *Laccaria laccata* and *Volvopluteus gloiocephalus* sporocarps and their corresponding underneath soils (CaCl_2_-extractable concentrations, except pseudo-total As and Pb). Coefficient of Pearson correlations (*r*) and probability value (*p*) are indicated for N = 10. Bold values indicate *p* < 0.05.

|  | *Laccaria laccata* | | *Volvopluteus gloiocephalus* | |
| --- | --- | --- | --- | --- |
|  | *r* | *p* | *r* | *p* |
| As | 0.56 | 0.09 | 0.42 | 0.23 |
| Cd | -0.21 | 0.55 | 0.38 | 0.28 |
| Co | **0.79** | **0.01** | 0.30 | 0.40 |
| Cu | -0.30 | 0.39 | -0.47 | 0.17 |
| Fe | -0.13 | 0.72 | 0.60 | 0.07 |
| Mn | 0.57 | 0.08 | 0.07 | 0.85 |
| Ni | -0.04 | 0.91 | -0.05 | 0.89 |
| Pb | 0.50 | 0.14 | 0.22 | 0.55 |
| S | 0.19 | 0.59 | -0.31 | 0.38 |
| Zn | 0.25 | 0.49 | -0.25 | 0.48 |

**Table Supplementary 2.** Contaminant provisional daily tolerable intake limits established by the Joint FAO/WHO Expert Committee on Food Additives (WHO, 2017) and minimum fungi daily intake of studied fruiting bodies to reach limits, expressed as daily kg of fungi fw (fresh weight) based on a person of 70 kg bodyweight. PMTDI (provisional maximum tolerable daily intake).

| Contaminant | PMTDI  (µg kg^-1^ bw) | *Laccaria laccata*  Daily limit (kg fw) | *Volvopluteus gloiocephalus*  Daily limit (kg fw) |
| --- | --- | --- | --- |
| As | 3* | 1.29 | 3.80 |
| Cd | 0.83 | 0.132 | 0.069 |
| Cu | 500 | 3.19 | 6.49 |
| Fe | 800 | 2.25 | 13.52 |
| Pb | 3.6* | 0.99 | 3.25 |
| Zn | 300-1000 | 1.30 | 2.96 |

*: Revoked limit. Not possible to establish a new intake that would be considered health protective.

**Table Supplementary 3.** Concentration of trace elements (in mg kg^-1^ dry weight) in fruiting bodies of *Laccaria laccata* and *Volvopluteus gloiocephalus* fungal species. Mean, SE and range values for N = 10. For comparison, the usual range values for edible mushrooms from unpolluted sites are indicated (in mg kg^-1^ dry weight) (Kalač, 2010) (n.a. = data non-available). The maximum allowed concentrations for Cd and Pb in wild edible mushrooms for human consumption (European Commission, 2015, 2014) have been converted to mg kg^-1^dry weight.

| **Trace element** | ***Laccaria laccata*** | | | |  | ***Volvopluteus gloiocephalus*** | | | | **Usual range from unpolluted sites** | **Maximum limit by EU regulation** |
| --- | --- | --- | --- | --- | --- | --- | --- | --- | --- | --- | --- |
|  | **Mean** | **SE** | **Min** | **Max** |  | **Mean** | **SE** | **Min** | **Max** |  |  |
| **As** | 1.36 | 0.19 | 0.26 | 2.25 |  | 0.518 | 0.075 | 0.203 | 1.035 | 0.5 – 5.0 |  |
| **Cd** | 3.02 | 0.45 | 1.45 | 6.01 |  | 8.86 | 0.88 | 4.82 | 12.67 | 1.0 – 5.0 | 14.0 – 17.3 |
| **Co** | 0.363 | 0.098 | 0.134 | 1.202 |  | 0.035 | 0.015 | 0.008 | 0.154 | < 0.5 |  |
| **Cu** | 132 | 6 | 105 | 159 |  | 63.1 | 4.4 | 53.7 | 98.4 | 20 - 100 |  |
| **Fe** | 212 | 25 | 116 | 344 |  | 53.0 | 2.0 | 44.4 | 62.5 | 50 - 300 |  |
| **Mn** | 29.4 | 8.7 | 15.1 | 106.1 |  | 26.1 | 0.8 | 21.2 | 30.0 | 10 - 60 |  |
| **Ni** | 0.631 | 0.114 | 0.289 | 1.370 |  | 0.260 | 0.033 | 0.087 | 0.395 | Traces – 15.0 |  |
| **Pb** | 1.94 | 0.20 | 1.13 | 3.48 |  | 0.757 | 0.103 | 0.346 | 1.404 | < 5.0 | 4.2 – 5.2 |
| **S** | 0.210 | 0.006 | 0.176 | 0.235 |  | 0.407 | 0.006 | 0.385 | 0.433 | n.a. |  |
| **Zn** | 178 | 9 | 129 | 219 |  | 111 | 2 | 104 | 125 | 25 - 200 |  |
|  |  |  |  |  |  |  |  |  |  |  |  |

**References**

European Commission, 2015. Commission Regulation (EU) 2015/1005 of 25 June 2015 amending Regulation (EC) No 1881/2006 as regards maximum levels of lead in certain foodstuffs, Official Journal of the European Union.

European Commission, 2014. Commission Regulation (EU) No 488/2014 of 12 May 2014 amending Regulation (EC) No 1881/2006 as regards maximum levels of cadmium in foodstuffs, Official Journal of the European Union.

Kalač, P., 2010. Trace element contents in European species of wild growing edible mushrooms: A review for the period 2000-2009. Food Chemistry 122, 2–15. doi:10.1016/j.foodchem.2010.02.045

WHO, 2017. Evaluations of the Joint FAO/WHO Expert Committee on Food Additives (JECFA) [WWW Document]. 84th JECFA. URL http://apps.who.int/food-additives-contaminants-jecfa-database/search.aspx (accessed 4.9.19).
